# New onset unusual Wegener’s granulomatosis associated with COVID-19: a case report

**DOI:** 10.1186/s43163-022-00370-3

**Published:** 2023-01-03

**Authors:** Mohammad Mandegari, Fariba Binesh, Mahsa Abdollahpour

**Affiliations:** 1 Department of Otolaryngology-Head and Neck Surgery, Shahid Sadoughi University of Medical Sciences, Yazd, Iran; 2 Department of Pathology, Shahid Sadoughi University of Medical Sciences, Yazd, Iran

**Keywords:** Wegener’s granulomatosis, Granulomatosis with polyangiitis (GPA), COVID-19, Autoimmune disorder, Vasculitis

## Abstract

**Background:**

Granulomatosis with polyangiitis (GPA) or Wegener’s granulomatosis is an autoimmune disorder with a wide spectrum of manifestations that mostly primarily presents with respiratory symptoms such as cough, dyspnea, and hemoptysis and leads to a high mortality rate if left untreated. It is a relatively uncommon condition, characterized by necrotizing granulomatous vasculitis of small- and medium-sized vessels. Recent studies have shown that hyperactivation of immune cells in patients with the coronavirus disease 2019 (COVID-19) leads to elevated levels of various autoantibodies and inflammatory cytokines including interferon-gamma (IFN-*γ*) and tumor necrosis factor-*α* (TNF-*α*). There are the same factors that involve in the pathogenesis of autoimmune diseases such as GPA.

**Case presentation:**

While there have been several reported cases of COVID-19 occurring in patients receiving immunosuppressant treatment for GPA, here we report a case of a 72-year-old woman with a history of coronavirus disease 2019 (COVID-19) who suddenly suffered unilateral vision and hearing loss and peripheral facial palsy on the same side. Chest computed tomography (CT) demonstrated a subpleural consolidation in the inferior lobe of the left lung. Based on the radiology report, chest CT evidence was due to a history of COVID-19 pneumonia. CT scans of the paranasal sinus showed pansinusitis and necrosis of the nasal septum. According to the available evidence, mucormycosis was clinically suspected, and the patient underwent endoscopic sinus surgery. Eventually, the histopathological analysis revealed a diagnosis of Wegener’s granulomatosis.

**Conclusions:**

Since GPA and its complications can be prevented only through strong clinical suspicion and early diagnosis, our presentation of this case aims to increase awareness of autoimmune diseases in COVID-19 patients even after recovery.

## Background

Granulomatosis with polyangiitis (GPA) is a rare rheumatologic disorder under the category of anti-neutrophil cytoplasmic antibody (ANCA)-associated vasculitis with an annual reported incidence of 11.3 patients per million [1]. Diagnosis of the disease is challenging due to its various clinical manifestations [2], primarily involving the upper and lower respiratory tract and characterized by necrotizing granulomatous vasculitis of small- and medium-sized vessels [3]. Delay in diagnosis or management of this condition may lead to significant morbidity or even mortality [4]. The age of symptom onset has a wide distribution, with a peak incidence between the ages of 41 and 68 years [5]. GPA is diagnosed based on clinical, radiologic, and positive ANCA serology and histopathologic evidence of necrotizing granulomatous inflammation [6].

## Case presentation

**Fig. 1:**
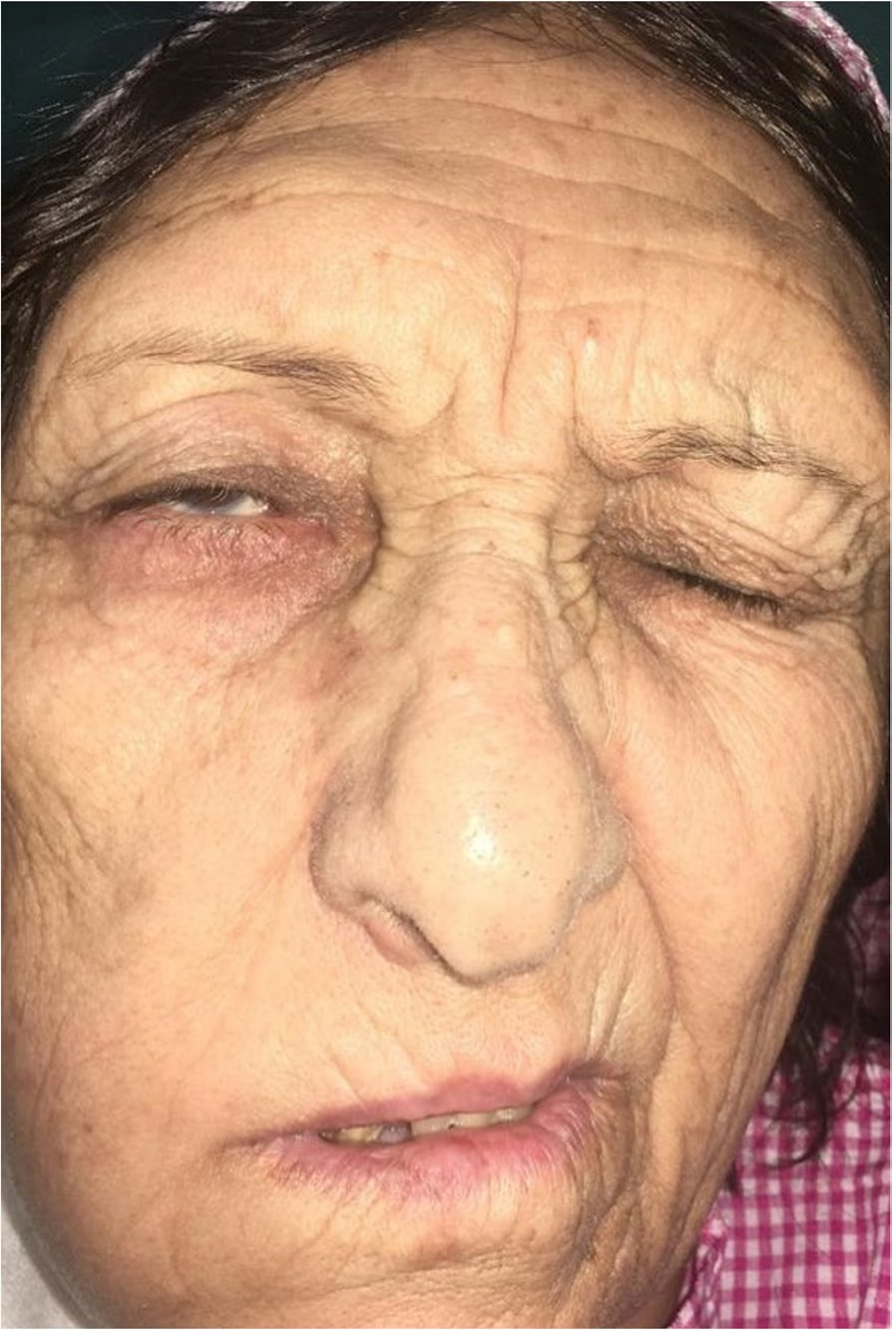
Right peripheral facial palsy (House–Brackmann score (HBS) grade 4)

**Fig. 2:**
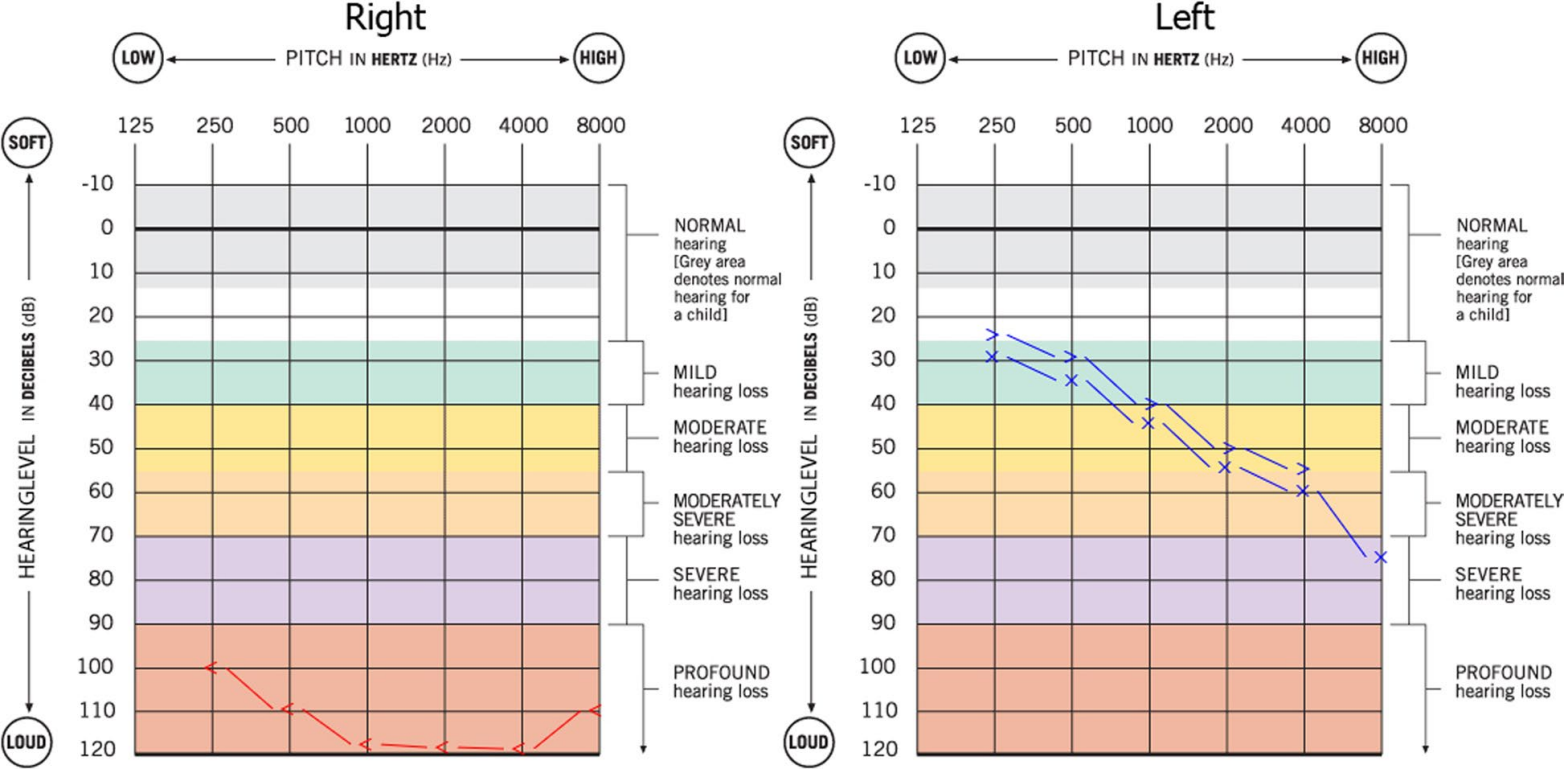
Pure tone audiometry test shows no bone conduction in the right ear

**Fig. 3:**
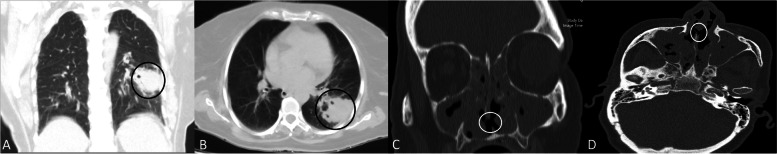
Radiological findings. **A** and **B** Coronal and axial computed tomography (CT) of the chest show the subpleural consolidation in the inferior lobe of the left lung (black circle). **C** and **D** Coronal and axial CT scans of the paranasal sinus show pansinusitis, necrosis, and perforation of the nasal septum (white circle)

**Fig. 4:**
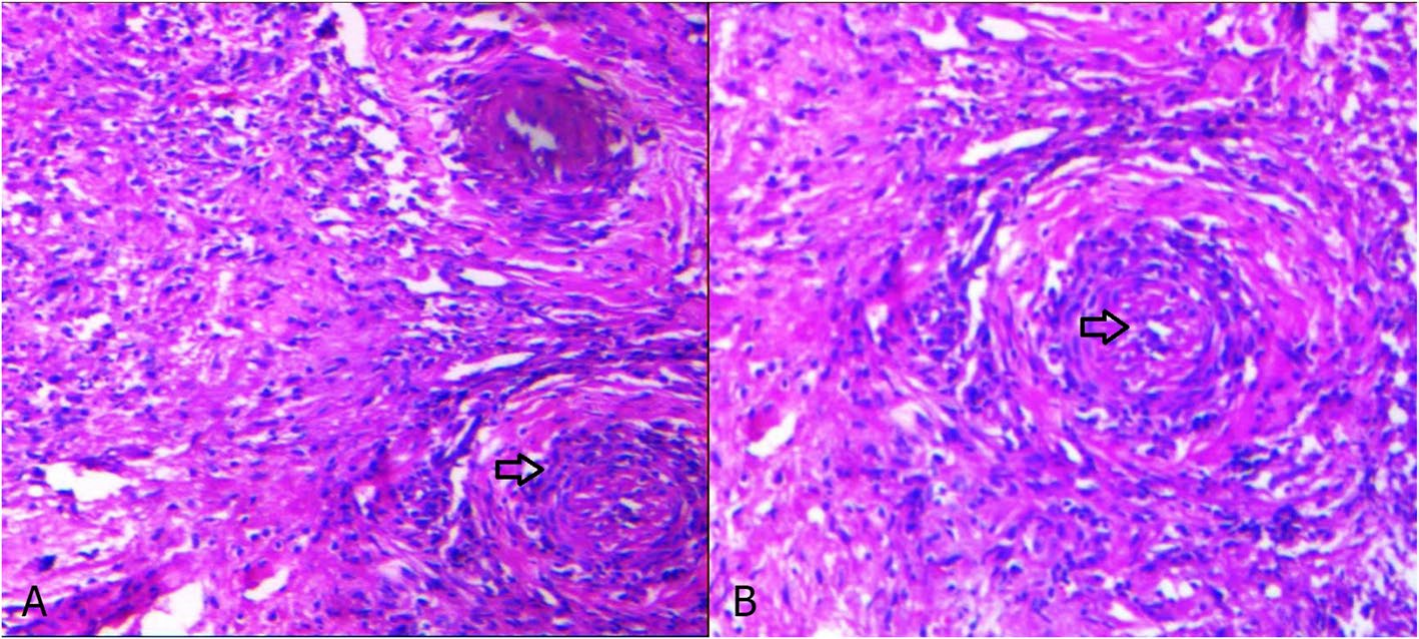
Histologic slide of the sinonasal biopsy specimen shows an area of necrosis that is bordered by granulation tissue which is infiltrated with numerous lymphocytes, plasma cells, and neutrophils (black arrow) (**A**); also, the vessel is involved with vasculitis (black arrow) (**B**)

A 72-year-old woman had a history of hypertension and ischemic heart disease, and there was no history of autoimmune disease. She was infected with the coronavirus disease 2019 (COVID-19) 2 months ago and presented with bilateral nasal congestion, rhinorrhea, and a headache. Since she refused to be hospitalized, her physician prescribed 50 mg (mg) of prednisolone and 400 mg cefixime per day and other symptomatic treatments as her outpatient care. While the patient’s symptoms were improving, she had to go to a local hospital 2 weeks later due to a persistent severe headache and sudden loss of vision in the right eye. On ophthalmologic examination, right central retinal artery occlusion (CRAO) was diagnosed immediately, and the patient underwent treatment with rivaroxaban (10 mg per day). Facial asymmetry on the right side and sudden ipsilateral hearing loss ensued, prompting her to present it to our hospital 10 days later. She was admitted to our center, and right peripheral facial palsy with House–Brackmann score (HBS) grade 4 was determined in clinical examinations Fig. 1. Ptosis was evident in the right eye with no light perception. Eye movement was normal, while there was ipsilateral relative afferent pupil defect (RAPD). No skin lesions were noted, and there was no numbness on the face, while rhinorrhea and facial pain and tenderness were evident on the right side. In the rhinoscopy, there was evidence of necrosis of the nasal septum and purulent secretions of the middle meatus. Otoscopic examination demonstrated central tympanic membrane perforation in the right ear. A pure tone audiometry test demonstrated there was no evidence of bone conduction (BC) in the right ear (Fig. 2). The lung examination revealed bilateral basilar dullness. All the aforementioned tests were normal on the left side. Severe acute respiratory syndrome coronavirus detection by real-time polymerase chain reaction (RT-PCR) using samples of nasopharyngeal revealed no sign of the COVID-19 infection. Serum C-reactive protein (CRP) level was severely elevated. The erythrocyte sedimentation rate (ESR) was increased up to 135 mm/h. Mild leukocytosis (13,000 cells/μl) with neutrophil dominance and normocytic anemia (hemoglobin level of 8.3 g/dl) and thrombocytosis (platelet count of 758,000/μl) was evident. Serum creatinine level was measured at 1.7 mg/dl with a urea level of 66 mg/dl. Liver enzymes were within normal range. The lactate dehydrogenase level was 355 IU/L. Iron studies revealed elevated ferritin and low iron. Rheumatoid factor, antinuclear antibody (ANA), and anti-citrullinated peptide antibody were assessed normally. Chest computed tomography (CT) demonstrated a subpleural consolidation in the inferior lobe of the left lung. Based on the radiology report, chest CT evidence was due to a history of COVID-19 pneumonia. CT scans of the paranasal sinus showed pansinusitis and necrosis of the nasal septum (Fig. 3). According to the available evidence, mucormycosis was clinically suspected, and the patient underwent endoscopic sinus surgery. On the endoscopic view, necrosis and perforation of the caudal and upper part of the nasal septum were confirmed. Necrotic debris and suspicious soft tissues were removed and underwent histopathological evaluation. Histopathologic analysis of the specimen showed necrotizing granulomatous inflammation and polyangiitis (formerly Wegener’s granulomatosis) (Fig. 4). Since this diagnosis was unexpected, we evaluated ANCA serology and performed a CT-guided biopsy of the subpleural consolidation of the left lung. Perinuclear antineutrophil cytoplasmic antibodies (P-ANCA) were negative, and cytoplasmic ANCA (C-ANCA) was positive (88.9 µ/ml). The lung specimen demonstrated the same histopathologic features of the diagnosis of GPA. The patient was referred to a rheumatologist, and after treatment with high-dose intravenous methylprednisolone 500 mg/dose given once daily for 3 days, she underwent medical treatments with prednisolone 1 mg/kg/day orally and methotrexate 15 mg/week orally. Now, 3 months after the beginning of medical treatment, other symptoms have improved and have not required any further hospitalizations, while there is no change in hearing and visual acuity.

## Discussion

Granulomatosis with polyangiitis is a multiorgan autoimmune disease with multifaceted clinical manifestations which, due to its rarity and the absence of distinctive clinical features, poses serious diagnostic challenges [2]. Diagnostic criteria for GPA consist of clinical evidence of disease in at least two of three areas (upper airways, lung, and kidney) and biopsy results showing disease at least once [7]. Some recent case reports introduced a possible relationship between coronaviruses and autoimmune disease. Zamani et al. reported a case that demonstrated a possible relationship between coronaviruses and systemic lupus erythematosus (SLE), and she suggested there are no published data on the association of coronaviruses with SLE, but several studies have linked coronaviruses with another autoimmune disease such as multiple sclerosis (MS) and rheumatoid arthritis (RA) [8]. Wegener’s granulomatosis or GPA is also an autoimmune disease where the pathogenesis of it is complex but consists of inflammatory cytokines [9]. Increased interleukin-17 (IL-17), interferon-gamma (IFN-γ), and tumor necrosis factor-alpha (TNF-α) levels lead to the formation of inflammatory granulomatous lesions in GPA [10]. Recent studies have shown that hyperactivation of immune cells in patients with COVID-19 leads to elevated levels of various autoantibodies and inflammatory cytokines including IFN-*γ* and TNF-*α* [11]. While there have been several reported cases of COVID-19 occurring in patients receiving immunosuppressant treatment for GPA [12, 13], in this study, we report new onset of Wegener’s granulomatosis with unusual manifestations in an aged woman 2 months after COVID-19 infection. Our patient did not have any history of autoimmune disease in her own or her family. After COVID-19 symptom’s recovery, she presented sudden unilateral visual and hearing loss accompanied by peripheral facial palsy and rhinosinusitis symptoms with clinical and radiological evidence of necrosis of the nasal septum, central tympanic membrane perforation, and there was no BC in pure tone audiometry test on the right side. Based on our experiences during the COVID-19 pandemic, in the encounter of an aged woman with a history of COVID-19 and glucocorticoid usage in the treatment of these sudden and rapidly progressive signs and symptoms aforementioned, primarily, we suspected mucormycosis. Eventually, the diagnosis of GPA was established via histopathological findings. Several studies have reported an increase in mucormycosis cases during the COVID-19 pandemic. COVID-19 and glucocorticoid usage as a basic treatment can lead to immune suppression in these patients and lead to the proliferation of opportunistic infections, including mucormycosis [14]. Mucormycosis is a rare but invasive, rapidly progressive, and life-threatening infection. A definite diagnosis of mucormycosis is only confirmed by histological findings. Early diagnosis of mucormycosis is so important given that a delay of diagnosis from 6 to 30 days can increase the mortality from 35 to 66% [15]. The most common clinical manifestation of mucormycosis is the involvement of the paranasal sinuses (39%), followed by the lung (24%), skin (19%), brain (9%), gastrointestinal tract (7%), and a disseminated type (6%), which is similar to GPA, in variety of manifestations, whereas the onset of symptoms in GPA is often more gradual [16]. COVID-19 and GPA share many clinical and radiological presentations. On chest CT, prominent findings in a GPA patient may be ground-glass appearance, consolidation, or even cavitation [12]. These findings may be mutual in a patient with COVID-19-superimposed pneumonia [17]. In our case, subpleural consolidation in the inferior lobe of the left lung was reported due to past COVID-19 pneumonia effects in radiological findings. Therefore, this evidence and normal oxygen saturation in our patient lead to the mass of the lung being an insignificant feature for the evolution of the correct identification. The diagnosis of GPA is often overlooked and delayed due to a wide range of clinical presentations. It is extremely rare to develop GPA after a COVID-19 infection. Jalalzadeh et al. reported ANCA-associated glomerulonephritis in a patient with systemic sclerosis after COVID-19 infection [18]. Bressler et al. reported a case of new-onset GPA with many skin manifestations in the setting of active COVID-19 infection [19]. Selvaraj et al. reported a 60-year-old woman who, 1 month after the COVID-19 infection, presented with persistent fatigue, shortness of breath, and anemia with worsening renal functions, found to have elevated C-ANCA and anti-proteinase 3 (PR3) antibodies, and was diagnosed with GPA [20]. But there is no published data about the new onset of Wegener’s granulomatosis with similar manifestations of our case after COVID-19 infection that has led to the development of serious diagnostic challenges for us.

## Conclusion

GPA or Wegener’s granulomatosis is an autoimmune disease that leads to high mortality rates if left untreated. Our case demonstrated once again that GPA presents a wide spectrum of manifestations and remains one of the most challenging diagnoses in clinical medicine. In this study, we reported a new onset of Wegener’s granulomatosis with unusual manifestations such as sudden unilateral visual loss and ipsilateral sensory neural hearing loss in an aged woman 2 months after COVID-19 infection. Some recent case reports introduced a possible relationship between coronaviruses and autoimmune disease. In addition, COVID-19 and GPA share many clinical and radiological presentations. In this case, subpleural consolidation in the lung was reported due to the history of COVID-19 pneumonia in radiological findings, while the lung specimen demonstrated the same histopathologic features as the GPA diagnosis. Since GPA and its complications can be prevented only through strong clinical suspicion and early diagnosis, it seems that physicians need to be aware of the autoimmune diseases in COVID-19 patients even after recovery.

## Data Availability

Not applicable.
